# Leaf water content contributes to global leaf trait relationships

**DOI:** 10.1038/s41467-022-32784-1

**Published:** 2022-09-21

**Authors:** Zhiqiang Wang, Heng Huang, Han Wang, Josep Peñuelas, Jordi Sardans, Ülo Niinemets, Karl J. Niklas, Yan Li, Jiangbo Xie, Ian J. Wright

**Affiliations:** 1 Sichuan Zoige Alpine Wetland Ecosystem National Observation and Research Station, Southwest Minzu University, 610041 Chengdu, China; 2 Institute of Qinghai-Tibetan Plateau, Southwest Minzu University, 610041 Chengdu, China; 3 Department of Environmental Science, Policy, and Management, University of California, Berkeley, Berkeley, CA 94720 USA; 4 Department of Biological and Agricultural Engineering, Texas A&M University, College Station, TX 77843 USA; 5 Department of Earth System Science, Ministry of Education Key Laboratory for Earth System Modeling, Institute for Global Change Studies, Tsinghua University, 100084 Beijing, China; 6 CSIC, Global Ecology Unit, CREAF-CSIC-UAB, Bellaterra, Catalonia 08193 Spain; 7 CREAF, Cerdanyola del Vallès, Catalonia 08913 Spain; 8 Institute of Agricultural and Environmental Sciences, Estonian University of Life Sciences, Kreutzwaldi 1, 51006 Tartu, Estonia; 9 Plant Biology Section, School of Integrative Plant Science, Cornell University, Ithaca, NY 14853 USA; 10 State Key Laboratory of Subtropical Silviculture, Zhejiang A&F University, 311300 Hangzhou, China; 11 Hawkesbury Institute for the Environment, Western Sydney University, Penrith, NSW 2750 Australia; 12 Department of Biological Sciences, Macquarie University, North Ryde, NSW 2109 Australia

**Keywords:** Plant ecology, Ecological modelling

## Abstract

Leaf functional traits are important indicators of plant growth and ecosystem dynamics. Despite a wealth of knowledge about leaf trait relationships, a mechanistic understanding of how biotic and abiotic factors quantitatively influence leaf trait variation and scaling is still incomplete. We propose that leaf water content (LWC) inherently affects other leaf traits, although its role has been largely neglected. Here, we present a modification of a previously validated model based on metabolic theory and use an extensive global leaf trait dataset to test it. Analyses show that mass-based photosynthetic capacity and specific leaf area increase nonlinearly with LWC, as predicted by the model. When the effects of temperature and LWC are controlled, the numerical values for the leaf area-mass scaling exponents converge onto 1.0 across plant functional groups, ecosystem types, and latitudinal zones. The data also indicate that leaf water mass is a better predictor of whole-leaf photosynthesis and leaf area than whole-leaf nitrogen and phosphorus masses. Our findings highlight a comprehensive theory that can quantitatively predict some global patterns from the leaf economics spectrum.

## Abstract (web summary)

Leaf functional traits are increasingly used as proxies for plant functions. Here, the authors show that leaf water affects other leaf traits and is a better predictor of whole-leaf photosynthesis and leaf area than leaf nitrogen or phosphorus content.

## Introduction

Leaf functional traits are closely related to plant growth and ecosystem dynamics^1,2^, and strongly influence carbon cycling and energy balance at the ecosystem level^3–6^. Several studies have explored the scaling relationships among leaf functional traits^1,7–12^. However, whether there exists a general model that quantitatively predicts all of the pertinent bivariate leaf trait relationships remains an open question. Among these important traits, leaf photosynthesis, specific leaf area (SLA), and leaf dry mass per area (LMA, the inverse of SLA) play critical roles because they reflect the potential for plant growth and mirror plant ecological strategies in response to environmental changes as well as along natural environmental gradients^3,13^. Considerable variations in leaf traits, such as SLA (and thus LMA), have been observed across plant species, functional groups, and environmental conditions^14^. Yet, the underlying mechanisms of these relationships remain elusive. In addition, variations in SLA indicate the variability of the scaling of leaf area (*A*_L_) with leaf dry mass (*M*_L_) which can be described by a generalised power law, i.e. ${A}_{{{{{{\rm{L}}}}}}}={{{{{\rm{\beta }}}}}}{{M}_{{{{{{\rm{L}}}}}}}}^{{{{{{\rm{\alpha }}}}}}}$, where α is the scaling exponent, and β is the normalisation constant. The overall numerical value of α is reported to be smaller than 1.0, indicating a phenomenon called “diminishing return” because increasing investments in *M*_L_ fail to produce proportional increases in *A*_L_. However, the numerical value of α can vary from <1.0 to >1.0 among or within some groups of species^8,10^. Nevertheless, theoretical attempts to explain the observed variability of α are rare and thus incomplete.

Tissue water content is another important, yet relatively under-investigated, plant functional trait. Water plays a fundamental role in biochemical reactions and metabolism^15^, and its availability regulates plant growth and metabolism, thereby influencing the productivity and carbon cycling of ecosystems^16–20^. At the leaf level, water availability may also directly affect other leaf traits, such as leaf photosynthetic rates and SLA (and thus LMA)^21^. In fact, leaf dry matter content (LDMC), which is functionally equivalent to leaf water content (LWC), has been suggested to be a better predictor of ecosystem above-ground net primary productivity (aNPP) than SLA^22^, although aNPP is influenced by many other factors^23^, suggesting an important role of LWC in the leaf economics spectrum, particularly since previous studies reveal that LWC plays a key role in leaf thermal regulation and further affects leaf carbon assimilation^24–26^. Therefore, exploring the quantitative links between LWC and other leaf traits could improve our understanding of not only trait variation and scaling relationships but also of trait shifts in response to climate change.

**Fig. 1:**
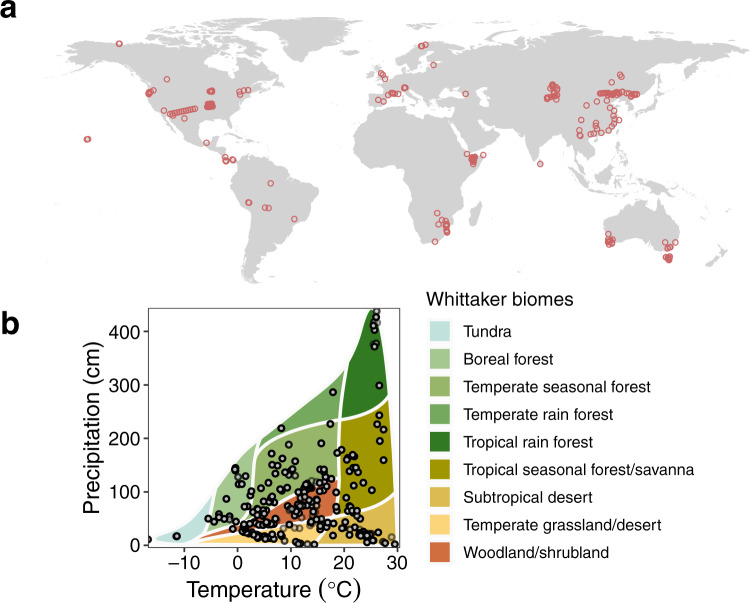
The distribution of the sampling sites of the studied leaf functional traits. **a** Global map showing the geographic locations of the field sites. **b** Two dimensional climate space represented by mean annual temperature and mean annual precipitation superimposed on Whittaker biomes.

In order to improve our understanding of these lineages, we derive a theoretical framework to explore the universal effects of temperature and LWC on leaf photosynthesis and SLA. We then use a comprehensive global leaf trait database compiled from 3427 species across a variety of ecosystems (Fig. 1) to (i) test whether the theoretical model successfully predicts the empirical relationships between leaf traits (mass-specific light-saturated leaf photosynthetic rate and SLA) and LWC and temperature and (ii) examine whether the exponent of leaf trait scaling converges onto a canonical value of 1.0 after temperature and LWC are corrected, as predicted by the model, across different plant life forms, ecosystem types, and latitudinal zones. Apart from LWC, leaf nitrogen (N) and phosphorus (P) are also considered two important traits in the leaf economics spectrum due to their essential roles in plant metabolism^27^ and also to their strong correlations with other leaf traits^1,10^. Thus, we further test the critical role of LWC by comparing the robustness of leaf water mass versus leaf nitrogen (N) and phosphorus (P) mass in predicting leaf photosynthesis and leaf area.

## Results and discussion

### The theoretical model

The model presented here builds on a recently developed metabolic theory based on biochemical kinetics. It describes a non-linear relationship between plant metabolic rate per unit of dry mass (*B*_s_, nmol g^−1^ s^−1^), such as light-saturated photosynthetic rates and dark respiration rates, plant water content (*S*, g g^−1^), and temperature (in degrees K)^19,20^, i.e.

1 $${B}_{{{{{{\rm{s}}}}}}}={g}_{1}{e}^{{k}_{1}S/\left({K}_{1}+S\right)}{e}^{-E/{kT}}$$

where *g*_1_ is a normalisation constant, *k*_1_ represents the maximum increase in specific metabolic rates due to changes in water content (i.e. from dehydrated to fully hydrated), *K*_1_ represents the water content when the mean reaction rate of cellular metabolism reaches one-half of its maximum, *E* is the activation energy, and *k* is Boltzmann’s constant. In this model *S* is defined on a dry mass basis (i.e. the ratio of plant water mass to plant dry mass) to broaden its range and better reflect the proportional changes in the amount of water in plant tissues^20^. Full details of the model’s assumptions can be found in the Methods and Huang et al.^19,20^. The model was tested and shown to hold true for a broad range of species and for whole plants and above- and belowground organs^20^. Here, we first applied the model to describe the quantitative effects of dry mass-based LWC (the ratio of leaf water mass to leaf dry mass) and temperature on the light-saturated leaf photosynthetic rate per unit of dry mass or leaf photosynthetic capacity (*P*_s_, nmol CO_2_ g^−1^ s^−1^), which can be expressed as

2 $${{{{{\rm{ln}}}}}}\left({P}_{{{\mbox{s}}}}\right)={{{{{\rm{ln}}}}}}\left(\frac{{P}_{{{{{{\rm{L}}}}}}}}{{M}_{{{{{{\rm{L}}}}}}}}\right)={{{{{\rm{ln}}}}}}\left({g}_{1}\right)+\frac{{k}_{1}\cdot {{{{{\rm{LWC}}}}}}}{{K}_{1}+{{{{{\rm{LWC}}}}}}}-\frac{E}{{kT}},$$

where *P*_L_ is the light-saturated whole-leaf photosynthetic rate (nmol s^−1^). Equation 2 indicates that the log-transformed temperature-corrected *P*_s_ (i.e. *P*_scor_) should increase with LWC, following Michaelis-Menten type hyperbolic response, i.e.

3 $${{{{{\rm{ln}}}}}}\left({P}_{{{\mbox{scor}}}}\right)={{{{{\rm{ln}}}}}}\left({P}_{{{{{{\rm{s}}}}}}}{e}^{E/{kT}}\right)={{{{{\rm{ln}}}}}}\left({g}_{1}\right)+\frac{{k}_{1}\cdot {{{{{\rm{LWC}}}}}}}{{K}_{1}+{{{{{\rm{LWC}}}}}}}.$$

Rearranging Eq. (2) shows that the temperature- and LWC-corrected whole-leaf photosynthetic rate (i.e. *P*_Lcor_) should scale isometrically with *M*_L_, i.e.

4 $${P}_{{{\mbox{Lcor}}}}={P}_{{{{{{\rm{L}}}}}}}{e}^{-{k}_{1}\cdot {{{{{\rm{LWC}}}}}}/\left({K}_{1}+{{{{{\rm{LWC}}}}}}\right)}{e}^{E/{kT}}={g}_{1}{M}_{{{{{{\rm{L}}}}}}}.$$

In this study, we refer to the temperature or water content correction as moving the temperature term ($\frac{E}{{kT}}$) or water content term ($\frac{{k}_{1}\cdot {{{{{\rm{LWC}}}}}}}{{K}_{1}+{{{{{\rm{LWC}}}}}}}$) to the left-hand side of the model, an approach that has been commonly used in previous studies^28^.

Following previous studies^29–31^, we assume that *P*_L_ is proportional to *A*_L_, i.e. ${P}_{L}\propto {A}_{L}$because leaf area directly determines the light interception capacity^1^. Therefore, the quantitative relationship between SLA and LWC and temperature can be described as

5 $${{{{{{\mathrm{ln}}}}}}}\left({{\mbox{SLA}}}\right)={{{{{{\mathrm{ln}}}}}}}\left(\frac{{A}_{{{{{{\rm{L}}}}}}}}{{M}_{{{{{{\rm{L}}}}}}}}\right)={{{{{{\mathrm{ln}}}}}}}\left({g}_{2}\right)+\frac{{k}_{1}\cdot {{{{{\rm{LWC}}}}}}}{{K}_{1}+{{{{{\rm{LWC}}}}}}}-\frac{E}{{kT}},$$

where *g*_2_ is another normalisation constant. Given that leaf area is a direct indicator of leaf photosynthetic capacity and that both traits reflect the long-term adaptation of plants to environmental change^3,13^, *k*_1_ in Eqs. (2) and (5) represent the maximum increase in mass-specific leaf photosynthetic capacity due to changes in water content. Since the temperature has a direct effect on the metabolic rates^32^ and productivity of ecosystems^33^, it is reasonable to assume that temperature can affect SLA globally^34,35^. Therefore, in this context *T* denotes the mean growing-season temperature (in degrees K), as SLA might be more responsive to long-term changes in temperature. Equation (5) predicts that SLA should increase with both LWC and the growing-season temperature. Rearranging Eq. (5) yields

6 $${{{{{{\mathrm{ln}}}}}}}\left({{\mbox{SL}}}{{{\mbox{A}}}}_{{{\mbox{cor}}}}\right)={{{{{{\mathrm{ln}}}}}}}\left({{\mbox{SLA}}}{e}^{E/{kT}}\right)={{{{{{\mathrm{ln}}}}}}}\left({g}_{2}\right)+\frac{{k}_{1}\cdot {{{{{\rm{LWC}}}}}}}{{K}_{1}+{{{{{\rm{LWC}}}}}}},$$

which predicts that the log-transformed temperature-corrected SLA (i.e. SLA_cor_) should increase with LWC following Michaelis-Menten dynamics. Likewise, by moving $\frac{{k}_{1}\cdot {{{{{\rm{LWC}}}}}}}{{K}_{1}+{{{{{\rm{LWC}}}}}}}$ and $\frac{E}{{kT}}$ to the left-hand side and *M*_L_ to the right-hand side of Eq. (5), we observe that the temperature- and LWC-corrected leaf area (i.e. *A*_Lcor_) should scale isometrically with leaf mass, i.e.

7 $${A}_{{{\mbox{Lcor}}}}={A}_{{{{{{\rm{L}}}}}}}{e}^{-{k}_{1}\cdot {{{{{\rm{LWC}}}}}}/\left({K}_{1}+{{{{{\rm{LWC}}}}}}\right)}{e}^{E/{kT}}={g}_{2}{M}_{{{{{{\rm{L}}}}}}}.$$

We note that the scaling of *P*_Lcor_ and *A*_Lcor_ with respect to *M*_L_ will reveal how LWC mediates the scaling exponent of leaf trait relationships.

### Effects of LWC and temperature on leaf trait scaling

**Fig. 2:**
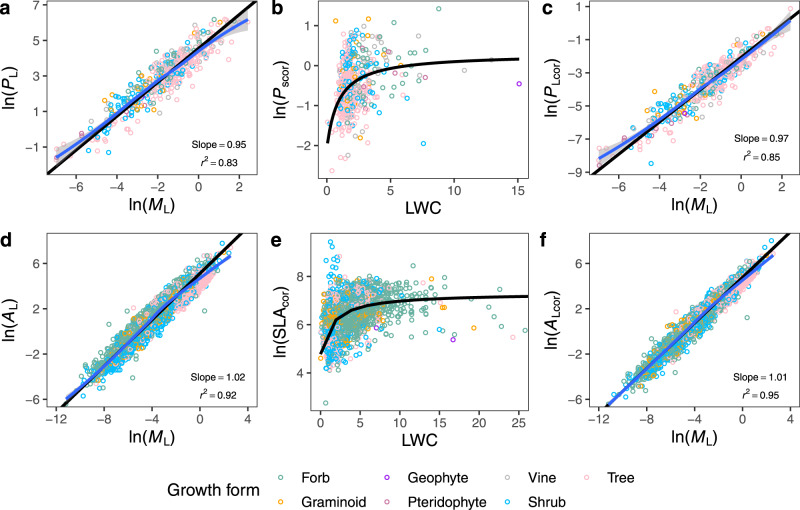
The quantitative effects of dry mass-based leaf water content (LWC) on leaf photosynthesis and SLA. **a** Scaling of leaf photosynthetic rate (*P*_L_, nmol s^−1^) with leaf dry mass (*M*_L_, g). **b** Non-linear fit to the relationship between temperature-corrected mass-specific leaf photosynthetic rate (*P*_scor_, nmol g^−1^ s^−1^) and LWC (g g^−1^) based on Eq. (3). **c** Scaling of temperature- and LWC-corrected leaf photosynthetic rate (*P*_Lcor_, nmol s^−1^) with *M*_L_ (g). **d** Scaling of leaf area (*A*_L_, cm^2^) with leaf dry mass (*M*_L_, g). **e** Non-linear fit to the relationship between temperature-corrected specific leaf area (SLA_cor_, cm^2^ g^−1^) and LWC based on Eq. (6). **f** Scaling of temperature- and LWC-corrected leaf area (*A*_Lcor_, cm^2^) with leaf dry mass (*M*_L_, g). Data with LWC greater than 25 were not shown in panel **e** for a better visualisation. LOWESS curves (blue lines) and 95% confidence intervals are shown.

The numerical value of the exponent for the *P*_L_ versus *A*_L_ scaling relationship calculated from the empirical data was 0.99 (Supplementary Fig. 1; 95% CI = 0.95 and 1.02, *r*^2^ = 0.87), strongly supporting the model assumption that *P*_L_ scales isometrically with *A*_L_. We then examined the effect of LWC on leaf trait scaling. The numerical value of the scaling exponent for the *P*_L_ versus *M*_L_ relationship was 0.95 (Fig. 2a; 95% CI = 0.92 and 0.99, *r*^2^ = 0.83). The non-linear relationship between *P*_scor_ and LWC described by Eq. (3) was supported by the empirical data (Fig. 2b; Supplementary Table 1). The non-linear model (Eq. 3) also had a lower Akaike’s Information Criterion score than the simple linear model between log-transformed *P*_scor_ and LWC (i.e. 793.5 versus 1988.8). After LWC and temperature were corrected (see Eq. 4), the numerical value of the scaling exponent became 0.97 (Fig. 2c; 95% CI = 0.94 and 1.01, *r*^2^ = 0.85), which was statistically indistinguishable from 1.0 (*P* > 0.05), as predicted by the model. Likewise, the numerical value of the exponent (i.e. α) for the *A*_L_ versus *M*_L_ scaling relationship was 1.02 (Fig. 2d; 95% CI = 1.02 and 1.03, *r*^2^ = 0.92). Additional analyses using the pooled dataset showed that log-transformed SLA increased with LWC following Michaelis-Menten dynamics, as predicted by Eq. (6) (Fig. 2e; Supplementary Table 1). After the effects of LWC and temperature were accounted for (using Eq. 7), the numerical value of α became 1.01 (Fig. 2f; 95% CI = 1.00 and 1.01, *r*^2^ = 0.95). Thus, both of the scaling exponents numerically converged onto 1.0 once LWC and temperature were corrected. In addition, an inspection of the locally weighted smoothing (LOWESS) curves showed that the curvature in both scaling relationships was reduced after the effects of LWC and temperature were corrected, as predicted by the model (Fig. 2).

The results presented here show that temperature and LWC quantitatively correlate with other leaf traits, such as *P*_s_ and SLA (or LMA), as predicted by the model. The increases in *P*_s_ and SLA attenuate with increasing LWC (Fig. 2b, e), indicating that leaf water availability sets a constraint on the maximum *P*_s_ and SLA that leaves can reach. It has long been recognised that SLA is closely correlated with leaf growth rate and metabolic activity^14,36,37^. Therefore, it is reasonable to also expect that SLA, as well as *P*_s_, will be quantitatively affected by LWC, which can change as a function of developmental status (such as leaf maturation and the accumulation of lignified tissues) and transiently as a function of evapotranspiration. LWC is also a reflection of species-specific adaptation to environmental conditions in different biomes. Nevertheless, our model, as well as the empirical data used to test it, reveal a broad and statistically robust correlation between critical leaf functional traits and leaf tissue water content. However, the observed variations in *P*_scor_ (Fig. 2b) suggest that in addition to temperature and LWC, other factors (e.g. plant phylogeny and soil fertility) may also affect leaf photosynthetic capacity, which is not accounted for in our model and should be critically examined in future research.

### Leaf area-mass scaling among different groups

**Fig. 3:**
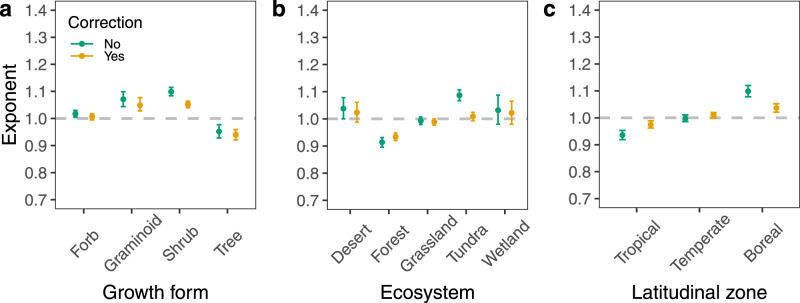
The exponents of leaf area-mass scaling with and without temperature and LWC corrections among different groups. **a** Comparison of scaling exponents among plant growth forms (*n* = 1688, 491, 1097, and 832 for forbs, graminoids, shrubs, and trees, respectively). **b** Comparison of scaling exponents among ecosystem types (*n* = 97, 1367, 1285, 1271, and 114 for deserts, forests, grasslands, tundra, and wetlands, respectively). **c** Comparison of scaling exponents among different latitudinal zones (*n* = 1111, 2113, and 910 for tropical, temperate, and boreal zones, respectively). Error bars indicate 95% confidence intervals.

The numerical value of α varied across different plant growth forms, ecosystems, and latitudinal zones (Fig. 3 and Supplementary Table 2). In particular, the leaf area versus mass scaling relationship showed a clear pattern along a latitudinal gradient (Fig. 3c and Supplementary Table 2). The numerical value of α decreased from 1.10 in boreal regions (95% CI = 1.08 and 1.12, *r*^2^ = 0.91) to 1.00 in temperate regions (95% CI = 0.99 and 1.01, *r*^2^ = 0.91), and to 0.94 in tropical regions (95% CI = 0.92 and 0.95, *r*^2^ = 0.91). However, after correcting for the effects of LWC and temperature, as predicted, α converged onto 1.0 across all different groupings, and the *r*^2^ values of the scaling relationships also increased (Fig. 3 and Supplementary Table 2).

Our analyses show that LWC also affects the numerical values of the exponents of leaf trait scaling relationships, which helps to explain why different works sometimes report significant differences in the exponents governing these relationships^8,10^. In particular, the numerical values of the scaling exponent governing the leaf area versus mass scaling relationship differ among different plant growth forms, ecosystems, and latitudinal zones (Fig. 3 and Supplementary Table 2), indicating that no invariant “scaling exponent” (i.e. α) holds true for the leaf area-mass scaling relationship. For example, in our dataset, α is significantly smaller than 1.0 in tropical regions (i.e. in keeping with a “diminishing returns” relationship in leaf area with respect to increasing leaf mass), close to 1.0 in temperate regions (i.e. a break-even relationship), and significantly larger than 1.0 in boreal regions (i.e. an “increasing returns” relationship). This shift in α along a latitudinal gradient may be associated with the different strategies to cope with variations in water availability, e.g. the high evapotranspiration rates in tropical regions may constrain increases in leaf area with increasing leaf mass, therefore resulting in diminishing returns, whereas, in boreal regions, the reduced water stress may enable plants to maximise leaf area to achieve relatively high photosynthetic capacities. Despite the variability in the numerical values of scaling exponents across different groups, the degree of curvature in these scaling relationships is reduced, and exponents converge onto unity after the effects of LWC and temperature are accounted for (Fig. 3), as predicted by the model (Eq. 7). This finding indicates that the variations in the exponent can, at least partially, be ascribed to the effects of LWC on SLA and the relative rate of increase in leaf area versus leaf mass. It is noteworthy that the numerical value of the scaling exponent for the *P*_L_ versus *M*_L_ relationship is also very close to 1.0, indicating the relatively weak effects of temperature and LWC on leaf photosynthesis-mass scaling. This may be partially attributed to the relatively limited number of data with concurrent LWC measurements and the adaptation of leaf traits to long-term temperature changes (see below for detailed discussion). Nevertheless, more measurements on LWC and leaf photosynthetic rates are needed to further test how LWC mediates leaf photosynthesis-mass scaling.

Given that LWC was weakly correlated with *M*_L_ (Supplementary Fig. 2, log-log slope = −0.01, 95% CI = −0.02 and 0, *r*^2^ < 0.01, *P* = 0.17), it is reasonable to assume that LWC is independent of *M*_L_ in order to derive the change in temperature-corrected *P*_L_ and *A*_L_ with respect to differences in *M*_L_ i.e. $\partial \left({P}_{{{{{{\rm{L}}}}}}}{e}^{E/{kT}}\right)/\partial {M}_{{{{{{\rm{L}}}}}}}={g}_{1}{e}^{{k}_{1}\cdot {{{{{\rm{LWC}}}}}}/\left({K}_{1}+{{{{{\rm{LWC}}}}}}\right)}$ and $\partial \left({{A}_{{{{{{\rm{L}}}}}}}e}^{E/{kT}}\right)/\partial {M}_{{{{{{\rm{L}}}}}}}={g}_{2}{e}^{{k}_{1}\cdot {{{{{\rm{LWC}}}}}}/\left({K}_{1}+{{{{{\rm{LWC}}}}}}\right)}$. These equations predict that increases in temperature-corrected *P*_L_ and *A*_L_ with respect to *M*_L_ should become faster with increasing LWC when LWC is relatively low, and achieve a relatively constant rate (plateau) when LWC is saturated, such that ${k}_{1}\cdot {{{{{\rm{LWC}}}}}}/\left({K}_{1}+{{{{{\rm{LWC}}}}}}\right)\approx 1$ (Supplementary Fig. 3). Therefore, LWC plays an essential role in regulating the dynamics of whole-leaf photosynthesis and area.

### Leaf water mass is a robust predictor of leaf photosynthesis and area

**Fig. 4:**
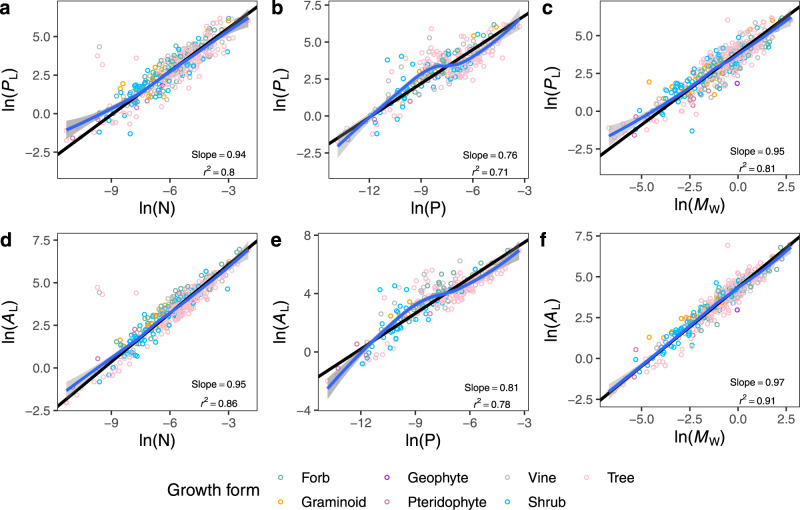
Leaf water is a robust predictor of leaf photosynthesis and area. The scaling of whole-leaf photosynthesis (*P*_L_, nmol s^−1^, **a–c**) and leaf area (*A*_L_, cm^2^, **d–f**) with leaf nitrogen (N, g), phosphorus (P, g), and water mass (*M*_W_, g). The SMA regression lines (black lines), the LOWESS curves (blue lines) and 95% confidence intervals (grey areas) are shown.

We used the China Plant Trait Database^38^ (see Methods) to explore whether whole-leaf N and P mass (g) or water mass (*M*_W_, g) show stronger scaling relationships with *P*_L_ and *A*_L_. This dataset was specifically used for this purpose because it includes paired data required for this comparison. The numerical values of the scaling exponents of the relationships of *P*_L_ versus leaf N and P were 0.94 (Fig. 4a; 95% CI = 0.90 and 0.98, *r*^2^ = 0.80) and 0.76 (Fig. 4b; 95% CI = 0.72 and 0.82, *r*^2^ = 0.71), respectively. Likewise, the exponents for the scaling of *A*_L_ versus leaf N and P were 0.95 (Fig. 4d; 95% CI = 0.92 and 0.99, *r*^2^ = 0.86) and 0.81 (Fig. 4e; 95% CI = 0.77 and 0.86, *r*^2^ = 0.78), respectively. However, *P*_L_ and *A*_L_ scaled as the 0.95 power (Fig. 4c; 95% CI = 0.91 and 0.99, *r*^2^ = 0.81) and as the 0.97 power of *M*_W_ (Fig. 4f; 95% CI = 0.94 and 0.99, *r*^2^ = 0.91), respectively. Thus, *M*_W_ provided a higher explanatory power in predicting *P*_L_ and *A*_L_ with an exponent closer to 1.0 than either leaf N or P. Moreover, an inspection of the LOWESS curves (blue lines in the graphs of Fig. 4) showed that the scaling of *P*_L_ and *A*_L_ with respect to both leaf N and P had a stronger degree of curvature than the scaling of *P*_L_ and *A*_L_ with respect to *M*_W_. Thus, water appears to have a higher hierarchical status than nutrients in terrestrial ecosystems where almost all biological activities, including nutrient uptake, depend on water availability^15^.

Leaf N and P are considered important leaf traits in the leaf economics spectrum because of their close correlations with other leaf traits, such as *P*_L_ and *A*_L_^1,9,10^. However, the numerical values of scaling exponents are known to vary significantly across different plant groups^9^, and no mechanistic model has been proposed to explain this variation. In this study, we found that the scaling relationships of *P*_L_ and *A*_L_, with respect to leaf N and P, exhibited curvature, whereas using *M*_W_ resulted in much more robust and linear scaling relationships (Fig. 4). These analyses indicate that leaf water availability may be a better indicator of *P*_L_ and *A*_L_ compared to leaf N and P, probably because (i) water serves as an essential biochemical reactant, solvent, and nutrient carrier in plants^15,20^, which indicates that leaf water availability plays a more fundamental role in determining leaf growth and metabolism than leaf N and P; and (ii) LWC is more sensitive to changes in endogenous (e.g. developmental age) and exogenous factors (e.g. drought stress) thereby providing a better reflection of plant growth status and changes in other leaf traits^15,39^. Leaf N shows a more robust scaling relationship with *P*_L_ than P (Fig. 4a, b), which is reasonable because N is a major component of key enzymes involved in photosynthesis, such as Rubisco.

### The important role of LWC in the leaf economics spectrum

Although LWC is a relatively understudied leaf trait, its important role in the leaf economics spectrum is indicated by the strong correlations between LDMC, a trait that is functionally related to LWC, and other traits, such as SLA^40^. Recent work reveals that LDMC also affects the ability of leaves to store and exchange heat in response to changes in surface energy fluxes, which further influences leaf carbon assimilation^26^. In addition, LDMC is predicted to be an effective indicator of plant resource use^41^ and ecosystem net primary production^22^, which corroborates the crucial role of LWC in regulating leaf growth and metabolism and, therefore, influencing productivity at the whole-plant and at the ecosystem levels. Although leaf functional traits have been extensively explored in ecological studies^1,7–12^, leaf trait variation across species or ecosystems, as well as the quantitative relationships among leaf traits, are not well understood due to the lack of a general model that captures the fundamental mechanism governing leaf trait variation. Our findings indicate that LWC is an important leaf trait that shows non-linear quantitative relationships with other important leaf traits, i.e. *P*_s_ and SLA (or LMA), and directly regulates the leaf trait scaling relationships. Although temporal variations in LWC might exist in response to instantaneous changes in microclimate conditions, previous work indicates that variation in LDMC (and thus LWC) is relatively conserved across seasons^42^. Further investigations should be conducted to better understand the temporal (e.g. seasonal and yearly) dynamics of LWC and its link to the dynamics of other leaf traits across different species and ecosystems.

### Relatively weak effects of temperature and precipitation

It is worth noting that the energy activation *E* in the Boltzmann factor is observed to be very close to 0 for both the leaf photosynthesis-mass scaling (−0.13, 95% CI = −0.40 and 0.15) and leaf area-mass scaling relationships (0.03, 95% CI = 0.01 and 0.05), which is consistent with previous work showing that both individual tree growth and ecosystem productivity are weakly correlated with growing-season air temperature^33,43^. This observed weak effect of temperature may be probably attributed to species-specific short-term phenotypic acclimation and long-term adaptation of plant photosynthetic traits to changes in ambient temperature^43,44^. However, as discussed elsewhere^33,43^, we used long-term mean air temperature for the SLA analyses, which may not effectively reflect the leaf temperature at which leaf traits were measured. Future investigations are needed to critically examine the effect of leaf temperature on leaf trait variations and trait scaling relationships.

Although the effect of precipitation was not directly quantified in our model, it might be partially accounted for by the effect of LWC because the plant water content is closely correlated with a variety of hydrological processes such as precipitation and evapotranspiration, soil water status, and plant growth^16,20^. Our analyses show that both LWC and SLA_cor_ are overall weakly correlated with mean annual precipitation (Supplementary Fig. 4), indicating that precipitation alone might not be able to fully explain leaf trait variation. In contrast, LWC shows a strong non-linear correlation with other leaf traits as predicted by our model and as shown by using a global dataset, demonstrating that LWC is an integrative trait that plays a critical role in mediating leaf trait variability.

**Fig. 5:**
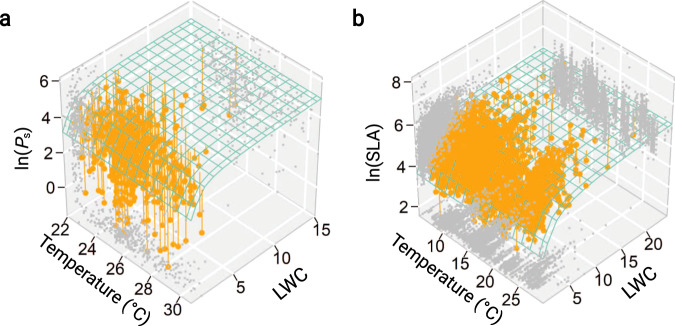
The three-dimensional leaf trait relationships. The relationships of log-transformed *P*_s_ (nmol g^−1^ s^−1^, **a**) and SLA (cm^2^ g^−1^, **b**) with temperature (°C) and LWC (g g^−1^). The dots projected on the floor and walls indicate bivariate correlations. The planes represent the theoretical predicted relationships based on Eqs. (2) and (5).

In this study, we combined a theoretical model and a global leaf trait dataset to quantify the effects of LWC and temperature on leaf trait scaling relationships (Fig. 5). The theoretical framework presented here provides a mechanistically deeper insight into the integrated functional traits of leaves and into how future changes in global climate may affect leaf traits such as *P*_s_ and SLA (and LMA). Empirical observations and global climate simulations predict an overall rise in temperature and especially an intensification of drought stress in many regions of the world^45,46^. These climatic changes will undoubtedly influence leaf traits, including *P*_s_ and SLA, directly through the effect of temperature or indirectly through the drought-induced changes in leaf structure and leaf water status. Our study highlights the importance of LWC as a simple but powerful trait that can be easily measured, and that quantitatively correlates with other important leaf traits. Consequently, LWC should be included in the leaf economics spectrum to better understand the trait variations and scaling relationships.

## Methods

### The model assumptions

In this study, we extended a previously published model^19,20^ to derive the model for leaf trait relationships. The previously validated model was developed to quantify the effects of body mass and temperature on plant metabolic rates based on the following assumptions. First, cellular metabolism is limited by the water availability of plant tissues, given the fundamental role of water in metabolism (e.g. as a biochemical reactant and nutrient carrier)^15^. In addition, the effect of tissue water content on the mean rate of metabolic chemical reactions follows the Michaelis-Menten type hyperbolic response. Finally, the rate of change in cellular metabolism is proportional to the rate of change in the critical metabolic chemical reaction. More details on the model derivation are provided in Huang et al.^19,20^.

### Data synthesis

To test the predictions of our model, we merged the TRY plant trait database^47^, the Tundra Trait Team (TTT) database^48^, the BROT plant functional trait database^49^, and the GLOPNET database^1^, and a database compiled from 12 additional peer-reviewed papers (see Supplementary Data sources). We focused on the six key traits most commonly reported across the dataset: leaf area, leaf fresh and dry mass, SLA, and LWC or LDMC. Data were included using two stringent criteria: (1) the data for leaf dry mass, leaf area, LWC or LDMC was concurrently measured and reported simultaneously; (2) only data from natural ecosystems at non-polluted sites were used (this excluded data from fertilised plants or plants grown in greenhouses). We also excluded data for which the identification of species was lacking. In total, the resulting dataset contained more than 17,000 trait measurements for a total of 3427 species distributed globally across different ecosystems (Fig. 1).

Given the relatively limited data available for instantaneous leaf photosynthetic rates with paired measurements of LWC, we used a specific dataset from the TRY plant trait database (i.e. the China Plant Trait Database^38^), which consists of paired leaf trait measurements (including leaf light-saturated photosynthesis rate, leaf area, LDMC, and leaf N and P) from 1215 plant species across a variety of ecosystems, to test the effect of leaf N and P mass versus leaf water mass on leaf photosynthesis and leaf area. We removed records with missing values of the selected leaf traits, resulting in a total of 404 measurements. Leaf mass-specific photosynthetic rate (nmol g^−1^ s^−1^) was converted from area-specific photosynthetic rate (umol m^−2^ s^−1^) by multiplying SLA (m^2^ kg^−1^). In this study, we used dry mass-based LWC (i.e. the ratio of leaf water mass to leaf dry mass, g g^−1^), which was calculated from the fresh mass-based leaf water content or LDMC reported in the global leaf trait dataset.

### Data analysis

For leaf photosynthetic rates, the in situ temperature at which the rates were measured was used for model fitting. For SLA, the monthly average temperature data (with a spatial resolution of ~1 km^2^) at each site were retrieved from the WorldClim 2.1 database (http://www.worldclim.org/). Following the previous work^1^, we defined “growing season months” as months with a mean temperature >4.99 °C. The growing-season temperature at each site was calculated as the average temperature across growing-season months. To reduce errors introduced by transient temperature changes, we used long-term temperature data to calculate growing-season temperatures. This “averaging” approach should be appropriate because of the great number of field sites included in this study. The mean annual precipitation (mm) at each site was also retrieved from the WorldClim 2.1 database to explore its correlations with LWC and SLA.

Given the differences in leaf sample sizes across sites, leaf trait values were averaged for each species at each sampling site to obtain species mean trait values. We note that we did not average the trait values for the same species across sites because some species exist in different ecosystems and latitudinal zones, which adds difficulty to the comparison analyses among ecosystem types and latitudinal gradients. Species were categorised into one of the four plant growth forms (trees, shrubs, graminoids, and forbs) and into one of the five ecosystem types (forest, grassland, desert, tundra, and wetland) to compare differences in the numerical values of scaling exponents among groups. Species belonging to geophytes, pteridophytes, and vines were only used for pooled data analysis due to their limited number of measurements. To investigate whether the numerical values of scaling exponents exhibited a latitudinal pattern, the data were segregated into three latitudinal zones (tropical 0–25°, temperate 25–50°, and boreal >50°). The bivariate scaling relationships among leaf traits for the pooled data and for each species group were fitted using standard major axis (SMA) regression, except for the scaling relationship between LWC and leaf mass, which was fitted using ordinary least squares (OLS) regression because of the expectation that the slope should be small and close to zero. These regression analyses were performed using the lmodel2 function in the R package lmodel2^50^. The leaf trait scaling relationships were also fitted by locally weighted sums of squares (LOWESS) smoothing lines to reveal the curvature across leaf size. The non-linear relationships of specific leaf photosynthetic rate and SLA with LWC and/or temperature *T* (Eqs. 2, 3, 5 and 6) were fitted to data by applying non-linear regression protocols using the nls function from the R package car (ver. 2.0–25). In the non-linear regression analysis, we treated 1/*kT* as a whole to estimate the activation energy *E* in Boltzmann’s factor. To obtain more robust estimates of model parameters, we applied the bootstrapping resampling approach for non-linear regression analysis with 1000 re-samplings using the function nlsBoot in the R package nlstools^51^. The three-dimensional plot was created using the scatter3D function in the R package plot3D^52^. All analyses were conducted using the statistical software R^53^.

### Reporting summary

Further information on research design is available in the Nature Research Reporting Summary linked to this article.

## Supplementary information

Supplementary Information

Reporting Summary

## Data Availability

The TRY plant trait database is publicly available at https://www.try-db.org. The TTT database is publicly available at https://github.com/TundraTraitTeam/TraitHub. The BROT plant functional trait database is publicly available at https://www.uv.es/jgpausas/brot.htm. The GLOPNET database is publicly available at https://www.nature.com/articles/nature02403 and also available from I.J.W. (ian.wright@mq.edu.au) upon request. The China Plant Trait Database is publicly available at 10.1002/ecy.2091. The WorldClim 2.1 database is publicly available at https://www.worldclim.org/.

## Code availability

The analysis was conducted using existing R packages, which are described in the “Methods”. The R codes for the data analysis are available from the corresponding author upon request.
