# Supplementary material for: Leaf water content contributes to global leaf trait relationships
Source: Nat Commun. 2022 Sep 21;13:5525. doi: 10.1038/s41467-022-32784-1 (PMC9492732; doi:10.1038/s41467-022-32784-1)
Supplement: Supplementary file 1 — Supplementary Information [file 41467_2022_32784_MOESM1_ESM.pdf]

Supplementary Information for

Leaf water content contributes to global leaf trait relationships

Zhiqiang Wang, Heng Huang*, Han Wang, Josep Peñuelas, Jordi Sardans, Ülo Niinemets,

Karl J. Niklas, Yan Li, Jiangbo Xie, and Ian J. Wright

*Corresponding author: Heng Huang, henghuang@berkeley.edu

Supplementary Table 1. Results of bootstrap nonlinear regression analysis by fitting the model to global leaf trait data.

Parameter	Estimate	S.E.	Low 95%CI	High 95%CI
Photosynthesis (Equation 2)				
$\ln(g_1)$	-2.25	5.40	-13.22	8.59
k_1	2.35	0.55	1.54	3.59
K_1	0.94	1.49	0.25	2.65
E	-0.13	0.14	-0.40	0.15
SLA (Equation 5)				
$\ln(g_2)$	4.79	0.36	4.07	5.48
k_1	2.53	0.07	2.40	2.68
K_1	1.52	0.16	1.23	1.86
E	0.03	0.01	0.01	0.05

Note: g_1 and g_2, intercepts; k_1, the maximum increase in $\ln(P_s)$ and ln(SLA) due to changes in leaf water content; K_1, leaf water content when the mean reaction rate of cellular metabolism reaches one-half of its maximum; E, activation energy.

Supplementary Table 2. Summary of leaf area-mass scaling relationships for the different plant groups.

Plant group	*n*	Temperature & LWC correction	Intercept	Exponent	95%CI	r^2
Growth form						
Graminoid	491	No	5.36	1.07	1.04-1.10	0.92
	491	Yes	5.04	1.05	1.03-1.07	0.93
Forb	1688	No	5.33	1.02	1.00-1.03	0.93
	1688	Yes	4.80	1.01	0.99-1.02	0.94
Shrub	1097	No	5.24	1.10	1.08-1.11	0.95
	1097	Yes	4.95	1.05	1.04-1.06	0.96
Tree	832	No	4.73	0.95	0.93-0.98	0.86
	832	Yes	4.54	0.94	0.92-0.96	0.91
Ecosystem						
Desert	97	No	5.07	1.04	1.00-1.08	0.97
	97	Yes	4.88	1.02	0.99-1.06	0.97
Grassland	1285	No	5.16	0.99	0.98-1.01	0.93
	1285	Yes	4.74	0.99	0.98-1.00	0.95
Forest	1367	No	4.91	0.91	0.90-0.93	0.87
	1367	Yes	4.68	0.93	0.92-0.95	0.92
Tundra	1271	No	5.29	1.09	1.07-1.11	0.89
	1271	Yes	4.68	1.01	0.99-1.02	0.92
Wetland	114	No	4.60	1.03	0.98-1.09	0.92
	114	Yes	4.49	1.02	0.98-1.07	0.95
Latitudinal zone						
Tropical	1111	No	4.99	0.94	0.92-0.95	0.91
	1111	Yes	4.65	0.98	0.96-0.99	0.94
Temperate	2113	No	5.05	1.00	0.99-1.01	0.91
	2113	Yes	4.81	1.01	1.00-1.02	0.94
Boreal	910	No	5.35	1.10	1.08-1.12	0.91
	910	Yes	4.91	1.04	1.02-1.05	0.95
All	4134	No	5.12	1.02	1.02-1.03	0.92
	4134	Yes	4.77	1.01	1.00-1.01	0.95

Supplementary Fig. 1. The scaling of temperature-corrected whole-leaf photosynthesis (P_L, nmol s^{-1}) with leaf area (A_L, cm^2).

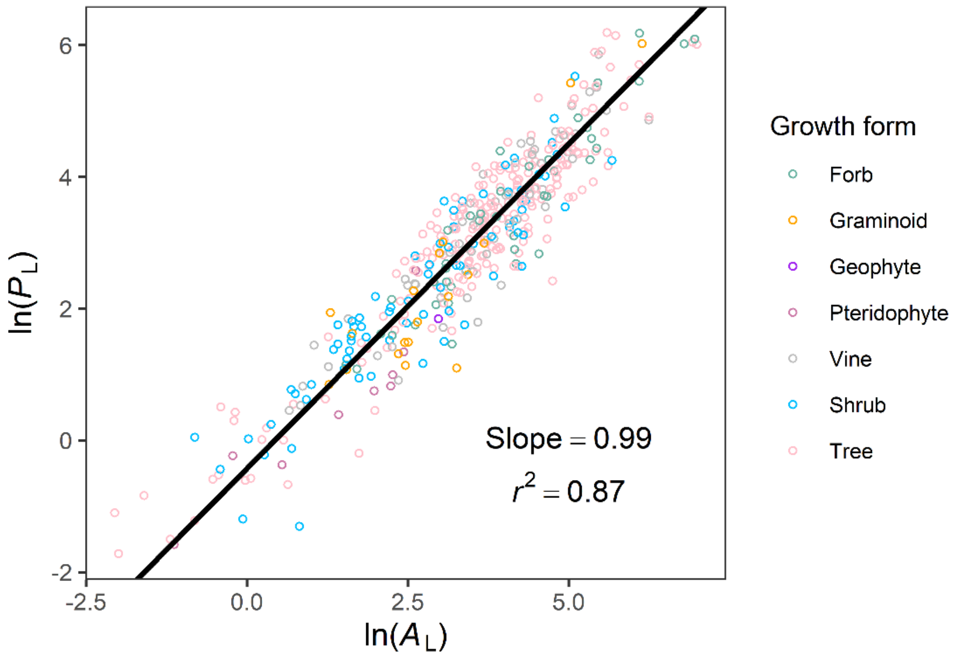

Supplementary Fig. 2. The scaling of leaf water content with leaf mass (M_L, g) across the pooled data.

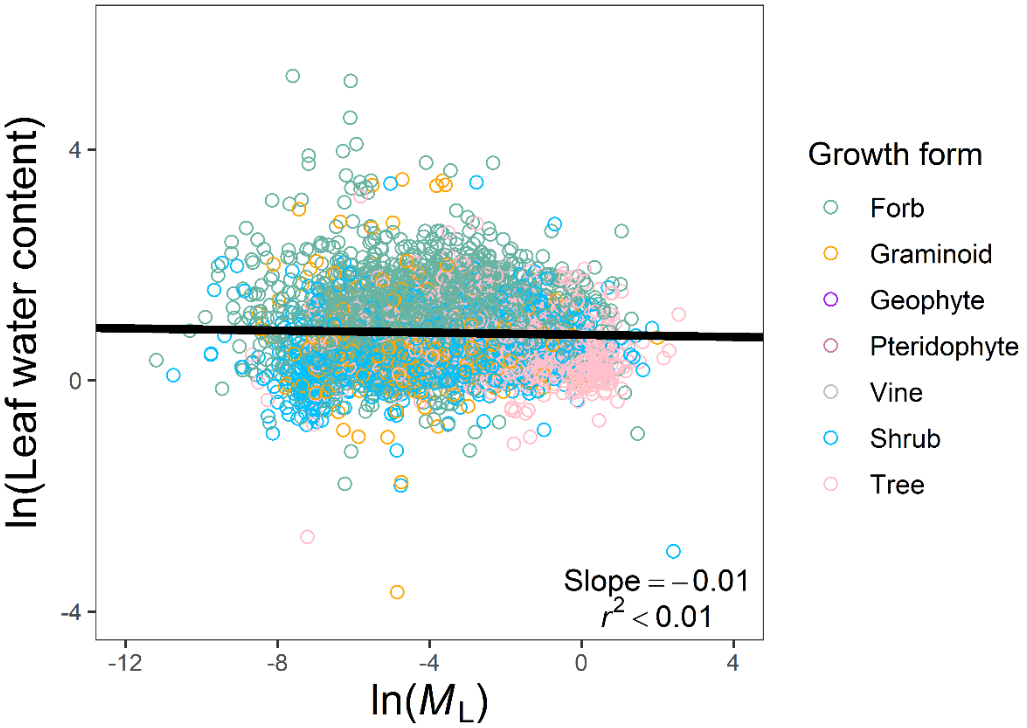

Supplementary Fig. 3. The effect of leaf water content on the rate of change in temperature-corrected leaf photosynthetic rate $P_{\mathrm{L}}e^{E/kT}$ (**a**) and leaf area $A_{\mathrm{L}}e^{E/kT}$ (**b**) with respect to differences in M_{L}. The numerical values of parameters k_1 and K_1 used to quantify the dynamics of $P_{\mathrm{L}}e^{E/kT}$ and $A_{\mathrm{L}}e^{E/kT}$ were obtained from the non-linear fitting results of Eqs. 2 and 5, respectively.

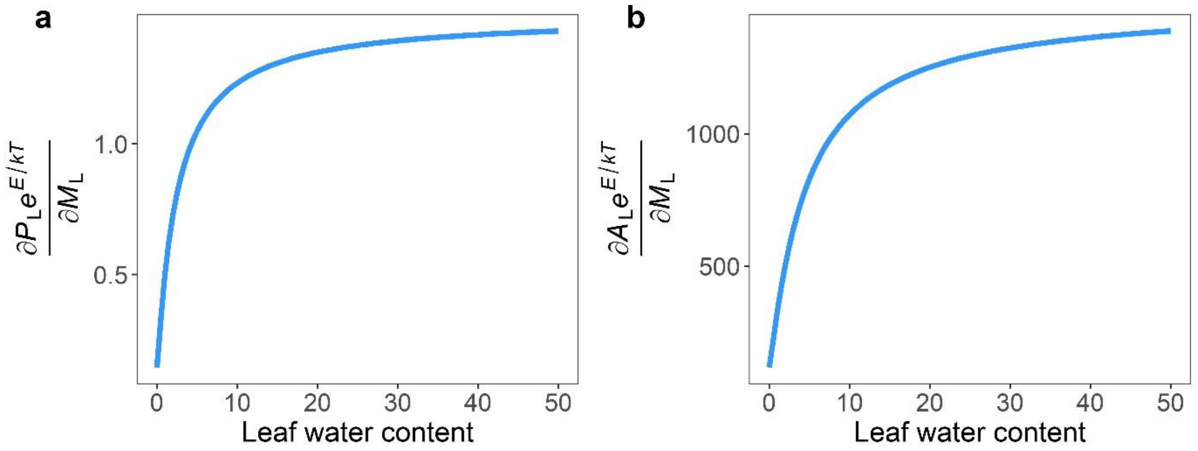

Supplementary Fig. 4. The scaling of LWC (**a**) and temperature-corrected SLA (**b**) with mean annual precipitation (MAP, mm) across the pooled data. LOWESS curves (blue lines) and 95% confidence intervals are shown.

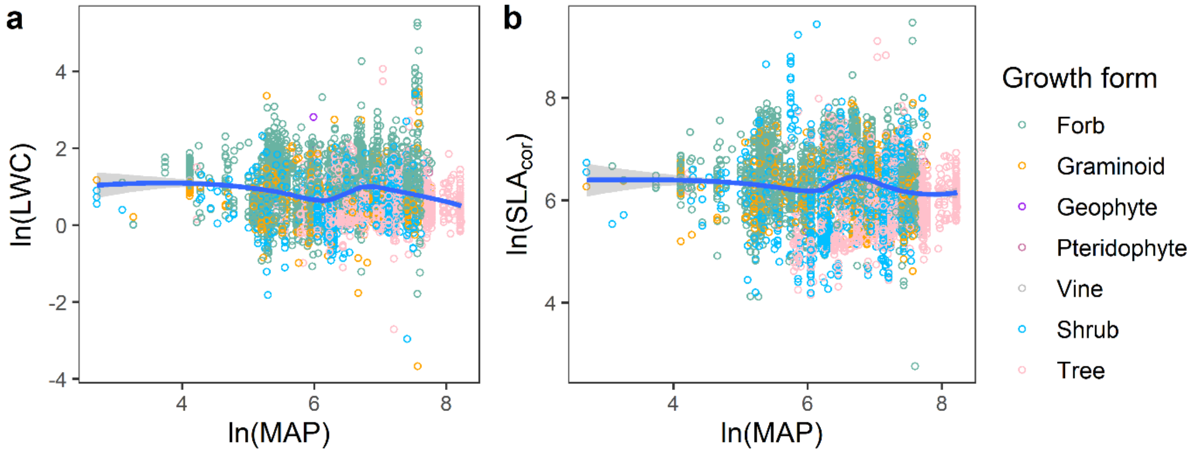

Data Sources

1. Anderegg, L. D. *et al.* Aridity drives coordinated trait shifts but not decreased trait variance across the geographic range of eight Australian trees. *New Phytol.* **229,** 1375-1387 (2020).
2. Bachmann, D., Roscher C. & Buchmann, N. How do leaf trait values change spatially and temporally with light availability in a grassland diversity experiment? *Oikos* **127,** 935-948 (2017).
3. Burton, J. I., Perakis, S. S., McKenzie, S. C., Lawrence, C. E. & Puettmann, K. J. Intraspecific variability and reaction norms of forest understory plant species traits. *Funct. Ecol.* **31,** 1881-1893 (2017).
4. Cerabolini, E. L. *et al.* Cand CSR classification be generally applied outside Britain? Plant *Ecology* **210,** 253-261 (2010).
5. De Long, J. R. *et al.* Relationships between plant traits, soil properties and carbon fluxes differ between monocultures and mixed communities in temperate grassland. *J. Ecol.* **107,** 1704-1719 (2019).
6. Hu, Y. K. *et al.* Is there coordination of leaf and fine root traits at local scales? A test in temperate forest swamps. *Ecol. Evol.* **9,** 8714-8723 (2019).
7. Miatto, R. C., Wright, I. J. & Batalha, M. A. Relationships between soil nutrient status and nutrient-related leaf traits in Brazilian cerrado and seasonal forest communities. *Plant Soil* **404,** 13-33 (2016).
8. Nunes, M. H., Davey, M. P. & Commes, D. A. Leaf trait variation and field spectroscopy of generalist tree species on contrasting soil types. *Biogeosciences* **14,** 3371-3385 (2016).
9. Soudzilovskaia, N. A. *et al.* Functional traits predict relationship between plant abundance dynamic and long-term climate warming. *Proc. Natl Acad. Sci. USA* **110,** 18180-18184 (2013).
10. Vandvik, V. *et al.* Plant traits and vegetation data from climate warming experiments along and 1100m elevation gradient in Gongga Mountains, China. *Sci. Data* **7,** 189 (2020).
11. Wigley, B. J. et al. Leaf traits of African woody savanna species across climate and soil fertility gradients: evidence for conservative versus acquisitive resource-use strategies. *J. Ecol.* **104,** 1357-1369 (2016).
12. Zhang, H. *et al.* Using functional trait diversity patterns to disentangle the

scale-dependent ecological processes in a subtropical forest. *Funct. Ecol.* **32,** 1379-1389 (2018).
