# Supplementary material for: Leaf water content contributes to global leaf trait relationships
Source: Nat Commun. 2022 Sep 21;13:5525. doi: 10.1038/s41467-022-32784-1 (PMC9492732; doi:10.1038/s41467-022-32784-1)
Supplement: Supplementary file 2 — Reporting Summary [file 41467_2022_32784_MOESM2_ESM.pdf]

nature portfolio

Corresponding author(s): Heng Huang

Last updated by author(s): Jul 30, 2022

Reporting Summary

Nature Portfolio wishes to improve the reproducibility of the work that we publish. This form provides structure for consistency and transparency in reporting. For further information on Nature Portfolio policies, see our Editorial Policies and the Editorial Policy Checklist.

Statistics

For all statistical analyses, confirm that the following items are present in the figure legend, table legend, main text, or Methods section.

n/a	Confirmed	
☐	☒	The exact sample size (*n*) for each experimental group/condition, given as a discrete number and unit of measurement
☐	☒	A statement on whether measurements were taken from distinct samples or whether the same sample was measured repeatedly
☒	☐	The statistical test(s) used AND whether they are one- or two-sided<br>*Only common tests should be described solely by name; describe more complex techniques in the Methods section.*
☐	☒	A description of all covariates tested
☒	☐	A description of any assumptions or corrections, such as tests of normality and adjustment for multiple comparisons
☐	☒	A full description of the statistical parameters including central tendency (e.g. means) or other basic estimates (e.g. regression coefficient) AND variation (e.g. standard deviation) or associated estimates of uncertainty (e.g. confidence intervals)
☒	☐	For null hypothesis testing, the test statistic (e.g. *F*, *t*, *r*) with confidence intervals, effect sizes, degrees of freedom and *P* value noted<br>*Give P values as exact values whenever suitable.*
☒	☐	For Bayesian analysis, information on the choice of priors and Markov chain Monte Carlo settings
☒	☐	For hierarchical and complex designs, identification of the appropriate level for tests and full reporting of outcomes
☒	☐	Estimates of effect sizes (e.g. Cohen's *d*, Pearson's *r*), indicating how they were calculated

Our web collection on statistics for biologists contains articles on many of the points above.

Software and code

Policy information about availability of computer code

Data collection	No software was used for data collection.
Data analysis	All analyses, including linear and non-linear regression analyses, were conducted using the statistical software R.

For manuscripts utilizing custom algorithms or software that are central to the research but not yet described in published literature, software must be made available to editors and reviewers. We strongly encourage code deposition in a community repository (e.g. GitHub). See the Nature Portfolio guidelines for submitting code & software for further information.

Data

Policy information about availability of data

All manuscripts must include a data availability statement. This statement should provide the following information, where applicable:

- Accession codes, unique identifiers, or web links for publicly available datasets
- A description of any restrictions on data availability
- For clinical datasets or third party data, please ensure that the statement adheres to our policy

The TRY plant trait database is publicly available at https://www.try-db.org. The TTT database is publicly available at https://github.com/TundraTraitTeam/TraitHub. The BROT plant functional trait database is publicly available at https://www.uv.es/jgpausas/brot.htm. The GLOPNET database is publicly available at https://www.nature.com/articles/nature02403 and also available from Dr. Ian Wright (ian.wright@mq.edu.au). The China Plant Trait Database is publicly available at https://doi.org/10.1002/ecy.2091. The WorldClim 2.1 database is publicly available at https://www.worldclim.org/.

Field-specific reporting

Please select the one below that is the best fit for your research. If you are not sure, read the appropriate sections before making your selection.

☐ Life sciences ☐ Behavioural & social sciences ☒ Ecological, evolutionary & environmental sciences

For a reference copy of the document with all sections, see nature.com/documents/nr-reporting-summary-flat.pdf

Ecological, evolutionary & environmental sciences study design

All studies must disclose on these points even when the disclosure is negative.

Study description	In this study, we used a general theory from first principles in biology to quantify the effects of temperature and leaf water content on other leaf traits and the corresponding scaling relationships. The theory was tested by existing leaf trait databases and literature. No treatments were involved in this study.
Research sample	In this study, all available data from the global leaf trait databases (including the TRY plant trait database, the Tundra Trait Team database, the BROT plant functional trait database, the GLOPNET database, the China Plant Trait Database, and peer-reviewed literature) were used. In total, the resulting dataset contained more than 17,000 trait measurements for a total of 3427 species distributed globally across different ecosystems.
Sampling strategy	We aim to compile a global leaf trait database based on available sources (TRY, TTT, BROT, GLOPNET, the China Plant Trait Database, and peer-reviewed literature). No sub-sampling was involved in this study.
Data collection	We obtained data from the TRY plant trait database, the Tundra Trait Team (TTT) database, the BROT plant functional trait database, the GLOPNET database, the China Plant Trait Database, and 12 additional peer-reviewed papers. The data collection procedures are generally not specified in the global databases. However, we screened the databases to ensure that the essential information (geographic location, species name, unit of traits) are available.
Timing and spatial scale	All available data from the global databases and peer-reviewed literature were included in this study, regardless of the sampling period. Since we focused on global leaf trait relationships, all data from across the globe were used.
Data exclusions	We excluded data from fertilised plants or plants grown in greenhouses. We also excluded data for which the identification of species was lacking in the global databases.
Reproducibility	All the data used in this study are publicly available and the associated links can be found in the manuscript. The analysis was conducted using existing R packages, which are described in the “Methods”. The R codes for the data analysis are available from the corresponding author upon request.
Randomization	Randomization was not involved in this study and analyses were conducted for different plant groups classified based on growth form, ecosystem type, and latitudinal zone.
Blinding	Blinding was not involved in this study because this is a plant functional trait study that analyzed all available data compiled across the globe.

Did the study involve field work? ☐ Yes ☒ No

Reporting for specific materials, systems and methods

We require information from authors about some types of materials, experimental systems and methods used in many studies. Here, indicate whether each material, system or method listed is relevant to your study. If you are not sure if a list item applies to your research, read the appropriate section before selecting a response.

Materials & experimental systems

n/a	Involved in the study	
☒	☐	Antibodies
☒	☐	Eukaryotic cell lines
☒	☐	Palaeontology and archaeology
☒	☐	Animals and other organisms
☒	☐	Human research participants
☒	☐	Clinical data
☒	☐	Dual use research of concern

Methods

n/a	Involved in the study	
☒	☐	ChIP-seq
☒	☐	Flow cytometry
☒	☐	MRI-based neuroimaging
